# Flavonoids in Cancer and Apoptosis

**DOI:** 10.3390/cancers11010028

**Published:** 2018-12-28

**Authors:** Mariam Abotaleb, Samson Mathews Samuel, Elizabeth Varghese, Sharon Varghese, Peter Kubatka, Alena Liskova, Dietrich Büsselberg

**Affiliations:** 1 Department of Physiology and Biophysics, Weill Cornell Medicine-Qatar, Education City, Qatar Foundation, Doha, P.O. Box 24144, Qatar; mariam.abotaleb@aucegypt.edu (M.A.); sms2016@qatar-med.cornell.edu (S.M.S.); elv2007@qatar-med.cornell.edu (E.V.); scv2002@qatar-med.cornell.edu (S.V.); 2 Department of Medical Biology, Jessenius Faculty of Medicine, Comenius University in Bratislava, 03601 Martin, Slovakia; kubatka@jfmed.uniba.sk; 3 Clinic of Obstetrics and Gynecology, Jessenius Faculty of Medicine, Comenius University in Bratislava, 03601 Martin, Slovakia; alenka.liskova@gmail.com

**Keywords:** anti-cancer therapy, apoptosis, cancer, flavonoids, natural compounds, phytochemicals

## Abstract

Cancer is the second leading cause of death globally. Although, there are many different approaches to cancer treatment, they are often painful due to adverse side effects and are sometimes ineffective due to increasing resistance to classical anti-cancer drugs or radiation therapy. Targeting delayed/inhibited apoptosis is a major approach in cancer treatment and a highly active area of research. Plant derived natural compounds are of major interest due to their high bioavailability, safety, minimal side effects and, most importantly, cost effectiveness. Flavonoids have gained importance as anti-cancer agents and have shown great potential as cytotoxic anti-cancer agents promoting apoptosis in cancer cells. In this review, a summary of flavonoids and their effectiveness in cancer treatment targeting apoptosis has been discussed.

## 1. Introduction

**Figure 1:**
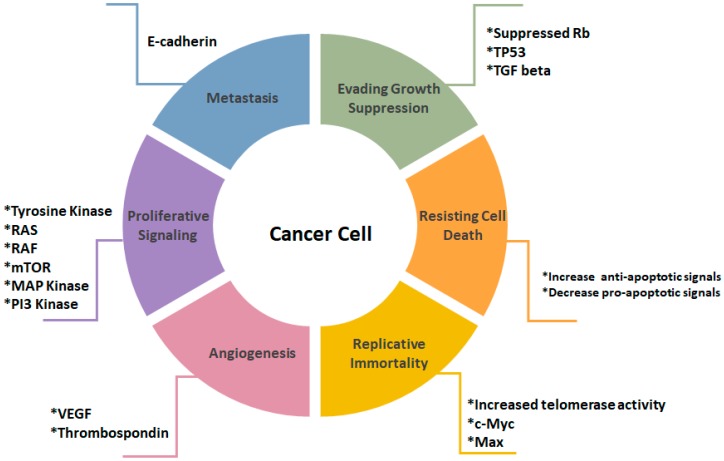
Illustration of cancer cell hallmarks. Evasion of growth suppression is mediated by inhibition of Rb, TP 53 and TGF beta. Resistance in cell death is signified by increase in anti-apoptotic and a decrease in pro-apoptotic factors. Replicative immortality is result of an increase in telomerase activity, c-Myc and Max. Angiogenesis is promoted due to the increase in VEGF and thrombospondin. The increased proliferative signaling is emphasized by increased tyrosine kinase activity, Ras, Raf, m-TOR, MAP kinase and PI3-kinase. Metastasis formation is marked by the loss of E-cadherin [4].

Cancer is a heterogeneous disease triggered by irreversible impairment of cellular homeostasis and function. Cancer progression is a result of uncontrolled cell growth and differentiation along with loss of apoptotic functions leading to a massive expansion in neoplastic cells population [1,2]. Internal causes of cancer may be attributed to lack of apoptotic function, genetic mutations, oxidative stress, and hypoxia, while external causes of cancer may be linked to excessive exposure to ultraviolet rays, radiation, pollution, smoking, and stress [3]. There are six hallmarks that contribute tumorigenesis and distinguish cancer cells from normal healthy ones (Figure 1) [4,5].

## 2. Apoptosis

Apoptosis is a stringently organized process, regulated by a series of signal transduction cascades and cellular proteins [6]. Cancer and many other diseases such as AIDS, diabetes, and Parkinson’s syndrome, occur as a result of imbalances and aberrant mechanisms in apoptotic pathways [7]. Thus, a profound understanding of apoptotic mechanisms and related pathways not only serves in comprehending the disease but also in disease treatment [8].

### 2.1. Apoptotic Proteins

**Figure 2:**
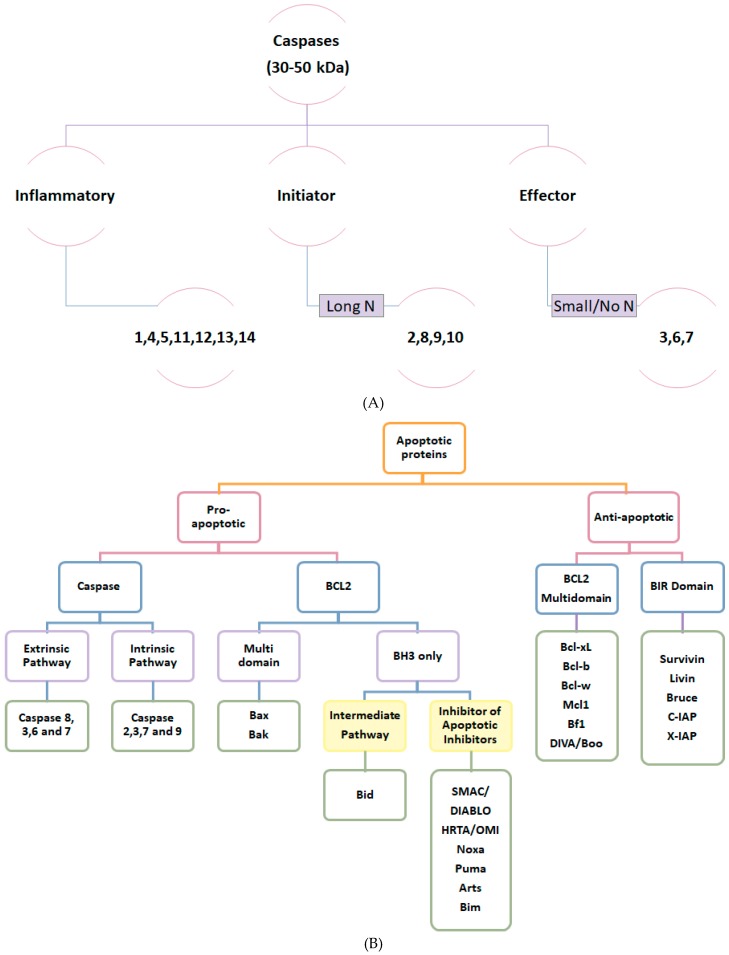
(**A**) Classification of caspases. Caspases are divided into three subtypes: inflammatory caspases (1,4,5,11,12,13,14), initiator caspases in intrinsic and extrinsic apoptotic pathways with long N terminal (2,8,9,10), and effector caspases known for their short/absent N-terminal (3,6,7) [6,8]. (**B**) Classification of apoptotic proteins according to their function in apoptosis. Pro-apoptotic proteins are classified into Caspases and BCL-2 families. Caspases are further subdivided according to their role in apoptotic pathway. Bcl-2 proteins are classified as multidomain proteins (Bax and Bak) and BH3 only domain that either serve in intermediate pathway (Bid and inhibitors of apoptotic inhibitors such as SMAC/Diablo, HRTA/Omi, Noxa, Puma, Arts and Bim). Anti-apoptotic proteins are classified as BCL-2 multi-domain proteins (Bcl-xl, Bcl-b, Bcl-w, Mcl-1, Bf1 and DIVA/Boo) and BIR domain family (survivin, livin, bruce, c-IAP and x-IAP).

Classification of apoptotic proteins depends primarily on their role in apoptosis (Figure 2B). Pro-apoptotic proteins are further classified depending on the protein family into (1) caspases and (2) Bcl2 family:

(1) Caspases, aspartate-specific cysteine proteases [1,6] possess a key role in apoptosis induction and are usually found as inactive heterodimers known as procaspases [6,9]. There are 14 known proteins belonging to caspases classified according to their structure and function (Figure 2A).

(2) Bcl-2 family (Figure 2B) regulates cells behavior through programmed cell death [8,10]. Localized to the outer membrane of the mitochondria, it mainly modulates the release of cytochrome c. Bcl-2 family of proteins is structurally distinguished by the presence of a BH3 domain [10]. Bcl-2 family either induces apoptosis via multi domain Bax/Bak or inhibits anti-apoptotic proteins (IAPs) belonging to BH3 only family including Puma, Noxa, Bim, Bad, Smac/Diablo, Hrta/Omi, Arts. Puma and Noxa which are crucial in cytokines induced apoptosis are produced as a result of DNA damage and transcription of P53 [2,6,11]. Bad (which is induced by nutrition deprivation and growth signals) [7,12], Smac/Diablo, Hrta/Omi, and Arts are all produced as a result of MOMP [7]. Anti-apoptotic proteins (Figure 2B), members of the Bcl-2 family functioning as apoptosis inhibitors, are subclassified into multi BH domain containing Bcl-xL, Bcl-B, Bcl-W, Mcl-1, Bf1 Diva/Boo. Under unstressed cellular conditions these proteins are upregulated in order to bind, interact, and sequester the pro-apoptotic Bax and Bak, preserving the mitochondrial membrane integrity [7,8]. BIR domain of anti-apoptotic proteins include N-IAP, c-IAP 1, c-IAP 2, and x-IAP. Testis specific survivin and ubiquitin conjugating enzymes livin and bruce/appollon all function mainly through the inhibition of caspases [7,8].

### 2.2. The Apoptotic Pathway

**Figure 3:**
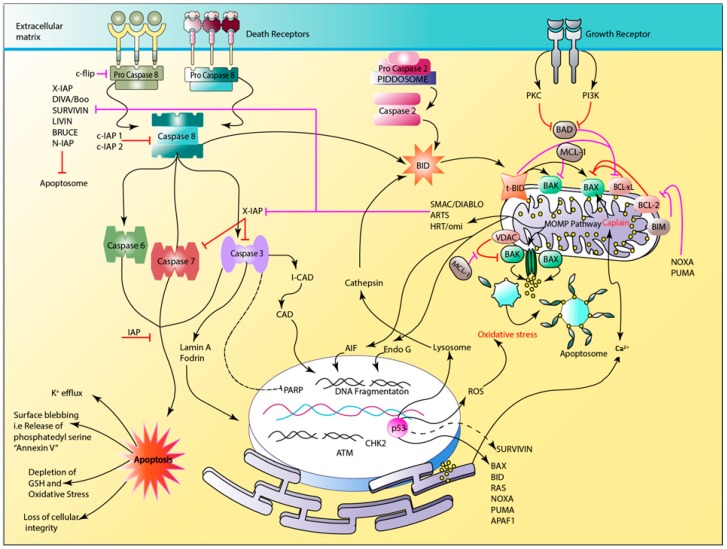
Illustration of intrinsic, extrinsic, mitochondrial, and endoplasmic reticulum apoptotic pathways. The role of apoptotic proteins (black arrows), and their inhibition by anti-apoptotic proteins (Red) which are regulated by apoptotic inhibitors (Pink). An apoptotic cell is usually characterized by increased K^+^ efflux, surface blebbing, depletion of anti-oxidative markers such as GSH, chromatin condensation, and loss of cellular integrity.

Two major pathways contributing to apoptosis are shown in Figure 3 [13]. Firstly, the extrinsic/death receptor induced pathway and secondly, the intrinsic pathway in which mitochondrial stress is involved. In extrinsic pathway the activation of signal transduction cascade occurs via binding of death signals to trimeric death ligands such as Fas and TNF [1,6]. Once an extra cellular death signal is received, initiator procaspases-8 and -10 (bound to the inner membrane FADD/TRADD regions of death receptors) are activated which results in the formation of a DISC complex and thus the signal transduction cascade proceeds [1,6,13]. Subsequently, activated caspase-3 functions in the activation of the mitochondrial amplification loop through the Bid protein containing BH3 only domain, thus serving as a link between extrinsic and intrinsic pathways [1,6,13]. t-Bid, an active form situated on the mitochondrial membrane, is responsible for the intrinsic cascade via the activation of pro-apoptotic multi-domain Bcl-2 proteins Bax and Bak [6]. Activated Bax and Bak mediate increasing MOMP through the formation of pores and change of the mitochondrial membrane potential (ΔΨm), resulting in the release of cytochrome c [2,7,9,13]. Cytochrome c, Apaf 1 protein, and procaspase-9 form an apoptosome, enhancing the apoptotic cascade through activation of executioner caspases [7].

Mitochondrial pores also facilitate the release of Smac/Diablo and Hrt/Omi which are BIR family inhibitors acting via inhibition of caspase-9 [14]. Intrinsic pathway could also be activated internally through oxidative stress, hypoxia, increased intracellular concentration of intracellular calcium ions, infection, or withdrawal of growth factors that obstruct the pro-apoptotic Bad protein that also stimulates Bax and Bak [7]. The activation of Bax and Bak is mediated through the inhibition of anti-apoptotic proteins Bcl-xL and Bcl-2 [6]. The function of Bak is restricted via VDAC. The activation of IAPs occurs as a result of the overexpression of tumor suppressor gene TP53 resulting in the activation of pro-apoptotic BH3 only domain proteins Bid (inhibiting Bcl-xL), Noxa (inhibiting Bcl-2) and Puma (inhibiting Mcl-1), all contributing to Bax-Bak pore formation and the release of cytochrome c. p53 functions in the process of ROS induction and increase of cellular stress as it was proven that ROS could enhance caspase function. ROS also stimulates H_2_O_2_ production, shifts the intracellular pH, and favors the activation of Bak. In addition, O_2_^−^ modulates the function of VDAC, hence increases mitochondrial membrane permeability [7]. p53 is also associated with lysosomal permeabilization, releasing cathepsin that activates Bid and Bak on the mitochondrial membrane [14,15]. Moreover, p53 is related to the activation of procaspase-2 through its release from p53-induced protein with a death domain PIDDOSOME. Subsequently, caspase-2 enhances the activation of t-Bid, boosting the mitochondrial amplification loop [15,16]. The MOMP pathway activates AIF and Endo G that increase DNA fragmentation [7,14]. Moreover, the release of Smac/Diablo inhibits the function of BIR domain family, preventing the inhibition of caspase 9 [7]. Cytochrome c factors in the release of Ca^2+^ ions through the activation of Inositol 1,4,5 phosphate on the surface of the ER to mitochondria resulting in accumulation of Ca^2+^ in mitochondrial space, hence increasing mitochondrial stress and pore formation [14,17]. Increased intracellular Ca^2+^ ion concentration also triggers the activation of calpain, an intracellular endonuclease that targets the decomposition of nuclear and cellular membranes [18,19]. In addition to function of caspase-3 in mitochondrial loop amplification, caspase-3 is also considered to be an executioner caspase, inhibiting and cleaving PARP associated with DNA repair process. PARP activates CAD which contributes to DNA fragmentation, as well as activation of Lamin A, resulting in chromatin condensation and nuclear membrane degradation. Inter alia, the formation of apoptotic bodies is realized after activation of fodrin [1,6]. Already mentioned reactions/pathways lead to characteristic physiological and morphological alterations such as cell shrinkage and blebbing (pyknosis), formation of apoptotic bodies, and membrane changes influenced by the phosphatidyl serine “Annexin V” allowing phagocytic cells to attack [8,9]. Accordingly, chromatin condensation (karyorrhexis), DNA fragmentation, breakdown of nuclear membrane, and increase of mitochondrial membrane permeability as well as high consumption of ATP are included in such alterations [1,8,9,18].

Cancer cells show resistance to apoptosis mediated by over expression of oncogenic genes (such as *c-Myc*, which enhances cellular proliferation and suppresses p53), and anti-apoptotic proteins (Bcl-2, survivin, livin, etc.) [9]. In contrast, the resistance of cancer cells to apoptosis can be also modulated via down regulation of pro-apoptotic proteins (caspases, Bad, Bax etc.) and the loss of tumor suppression function of p53 [1,7]. Overcoming resistance in apoptotic pathways is an interesting approach in cancer treatment [7]. This is mainly achieved through targeting p53 (drug, gene and immunotherapy), caspases (drug and gene therapy) and BIR family, and stimulating the action of IAP. Recently, plant-based treatment of cancer has been a highly active area of research across the globe. In this review, effects of flavonoids in cancer treatment and their influence on apoptotic pathways will be discussed.

## 3. Phytochemicals

### 3.1. Classification of Phytochemicals

**Figure 4:**
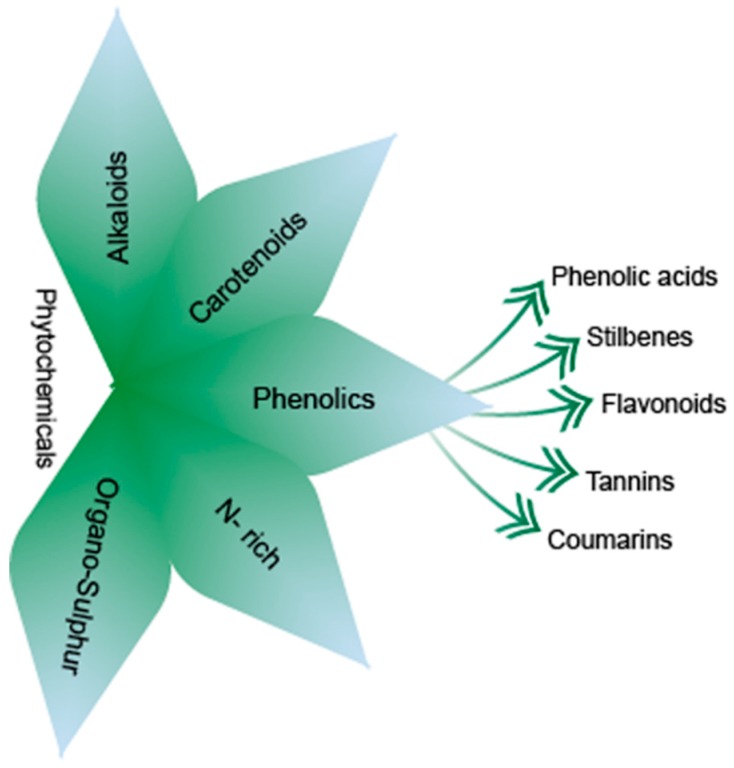
Classification of phytochemicals. alkaloids, carotenoids, n-rich, organo-sulphur compounds, and phenolics, which are further classified as phenolic acids, stilbenes, flavonoids, tannins, and coumarins.

Phytochemicals are bioactive non-nutrient compounds widely distributed in plants with an ability to reduce the risk of various diseases [20,21]. There are more than 50,000 different known phytochemicals in plant kingdom. The basic classification of phytochemicals is provided in Figure 4.

Significantly, phenolics possess one or more aromatic benzene ring and could be mono or poly hydroxylated. Moreover, phenolics are considered to be the most abundant antioxidants in human diet. Phenolics are classified as non-flavonoids including phenolic acids, stilbenes, tannins, coumarins, and flavonoids [22].

### 3.2. Flavonoids

**Figure 5:**
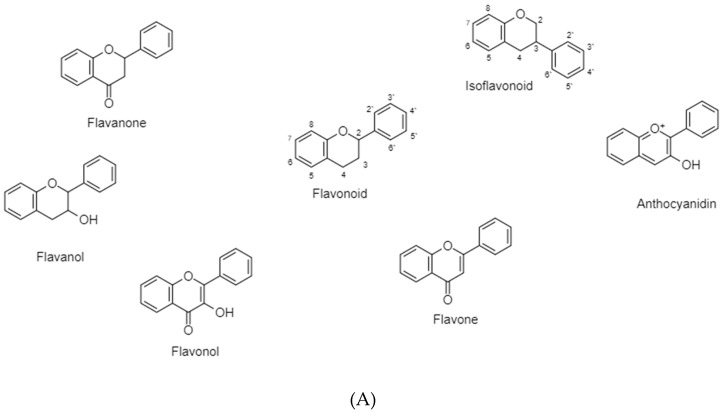

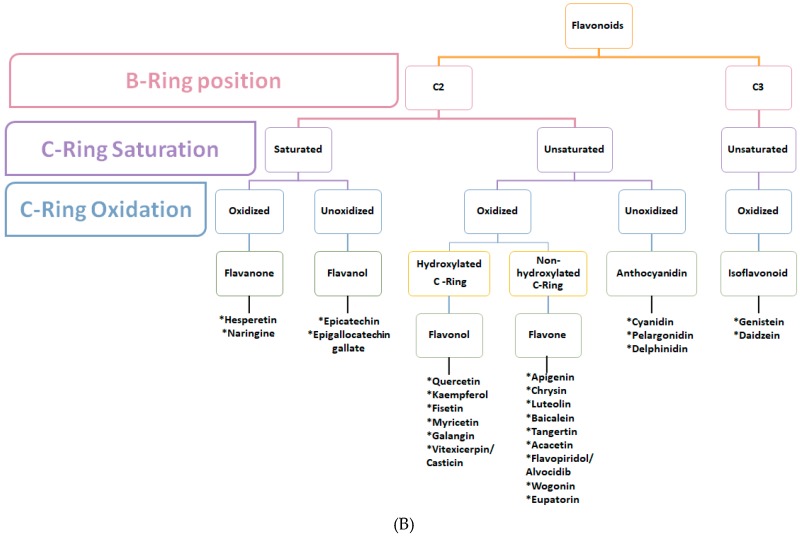
(**A**) Classification of flavonoids into six classes depending on structural differences: flavonol, flavanone, flavanol, flavone, anthocyanidin, and isoflavonoid. (**B**) Structural classification of plant flavonoids according to the position of B-ring on C2 or C3, B-ring saturation and oxidation.

Flavonoids are secondary plant metabolites responsible for color and aroma of flowers, and they possess antibacterial, antiviral, antioxidant, anti-allergic, and anti-inflammatory abilities [20,23,24]. In the process of carcinogenesis, flavonoids interfere with multiple signal transduction pathways and thus limit proliferation, angiogenesis, and metastasis or increase apoptosis [25] (Figure 5A,B).

Polyphenolic terpenoids consist of 15 carbon atoms arranged as a chromane ring (A and C) linked to a B ring in either C2 (Flavonoids) or C3 (isoflavonoids) [20,23]. As illustrated in Figure 5A,B, they could be further classified according to the C ring saturation and oxidation into 5 major classes [20,24,25]. Each class differs in the degree of hydroxylation and substitution [22]. Flavonoids exist either as glycosides with attached sugars or as aglycones with no attached sugars [26].

#### 3.2.1. Flavonoids (B Ring Attached to C2)-Saturated C-Ring

##### 3.2.1.1. Flavanone

**Figure 6:**
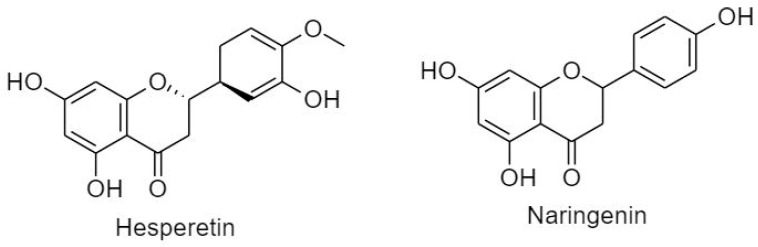
Flavanones. Hesperetin and naringenin.

Flavanones, also known as di-hydroflavones, are characterized by a saturated, oxidized C ring. Flavanones are distributed in citrus fruits and are known for their antioxidant activity and free radical scavenging ability [20]. Hesperetin and naringenin are two main flavanone compounds (Figure 6).

(A) Hesperetin

Hesperetin or 5,7,3′-trihydroxy-4′-methoxyflavanone is an aglycon present abundantly in citrus fruits, cherries, and tomatoes. Hesperetin and its glycoside, hesperidin, are known for their neuroprotective, vascular, anti-inflammatory, anti-allergic, antioxidant, and anticarcinogenic effects [27,28]. Hesperetin exerts anticancer abilities either by elevating ROS, attenuating mitochondrial potential, inducing DNA fragmentation, or by over expressing Fas and FADD ligands [29]. Pro-apoptotic effects of hesperetin were evaluated by both in vitro and in vivo studies. Hesperetin induced expression of cytochrome c, APAF-1, caspases-3 and -9 and reduced Bax to Bcl-2 ratio in gastric cancer cells [29]. Hesperetin was also found to induce cellular cell death in Eca109 cells by increasing ROS and decreasing GSH concentrations, confirming intrinsic mitochondrial cell death [30]. Similar pro-apoptotic effects were related to HT-29, MCF-7, and MDA-MB-231 cells [31,32]. Moreover, in the HT-20 cell line, hesperetin decreased the concentration of enzymatic antioxidants such as SOD, CAT, and GPx [32]. Conversely, in H522 cells, hesperetin induced apoptosis extrinsically via overexpression in Fas and FADD ligands, increase in caspase-9, and downregulation of p53 levels [33]. Furthermore, hesperetin was found to induce intrinsic and/or extrinsic apoptosis and cell cycle arrest at G2/M phase in the SiHa cell lines [34]. In addition to the inhibition of NF-κB pathway, hesperetin reduced Bcl-2 transcription and translation in PC-3 cell lines [35].

(B) Naringenin

5,7,4′-trihydroxyflavanone, also known as naringenin, is an aglycone found mostly in oranges and grapefruits. It has a broadly remarkable pharmacological profile and a wide range of biological effects, including anticancer, antioxidant, antimutagenic, antiproliferative, and anti-inflammatory abilities [36]. Anticancer properties of naringenin are induced either by elevating ROS, reducing mitochondrial potential, inducing DNA damage, or extrinsically by overexpression of Fas and FADD ligands. In a study conducted on SGC-7901 cells, naringenin induced apoptosis via increase in p53 expression, PARP cleaving, Bax and caspase-3 cleaving, and also decrease in the expression of Bcl-2 and survivin [37,38]. Moreover, in A431 and HepG2 cells, naringenin arrested cell cycle at G_0_/G_1_ and/or G_2_/M phase, decreased ΔΨm, and increased the caspase-3 or Bax/Bcl-2 ratio. Considering HepG2 cells, in vivo studies revealed that naringenin increased cytochrome c and AIF/Endo G mobilization from the mitochondrial membrane, and reduced GSH concentration, SOC and, catalase activity [39,40,41]. Similarly, naringenin treatment of MCF-7 cell lines resulted in activation of caspase-3 [42]. Furthermore, a naringenin synthesized derivate (N101-2) induced cell cycle arrest at sub-G_1_ phase in SiHa cells. Accordingly, intrinsic apoptosis was modulated due to elevated levels of p53, caspases-3, -8, and -9; increased PARP; and upregulation of Bax. On the contrary, extrinsic apoptotic pathway was confirmed by overexpression of Fas/FasL [43]. In HeLa and PC-3 cells, naringenin increased intracellular ROS level and induced loss of ΔΨm [44,45].

##### 3.2.1.2. Flavanols

**Figure 7:**
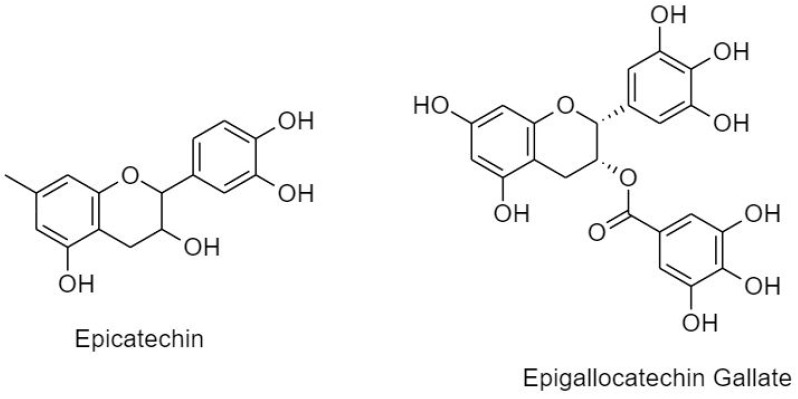
Classification and structure of flavanols.

Also known as catechins, flavan-3-ols are profoundly distributed in bananas, apples, peaches, pears, and blueberries [20]. Similar to flavanone, flavanol is characterized by a saturated C ring. However, it is usually unoxidized and possesses a hydroxyl group at C3. Catechins can exert cell cycle arrest via modulation of multiple signaling pathways such as nuclear factor-κB, MAPK, epidermal growth factor, vascular endothelial growth factor, and matrix metalloproteinase. Flavanols are subdivided into epicatechin and epigallocatechin gallate (Figure 7).

(A) Epicatechin

Epicatechin is widely distributed in dark chocolate, grapes, and green tea. Epicatechin is known to be a potent antioxidant contributing to apoptosis and preventing angiogenesis by modulation of signaling enzymes activity Antiproliferative properties of epicatechin were observed in both HeLa and MKN-45 cells in which H_2_O_2_-mediated oxidative stress led to the rapid destruction of cancer cells [46]. The findings of other studies suggested that epicatechin is also able to target proliferative signaling Akt and NF-κB. Interestingly, epicatechin in combination with panaxadiol or cisplatin showed synergistic pro-apoptotic effects in HCT-116 or renal tubular carcinoma respectively [47].

(B) Epigallocatechin Gallate (EGCG)

EGCG has been reported to induce apoptosis in many cancer cell lines. In prostate carcinoma cells EGCG considerably induced TRAIL-mediated apoptosis and, similarly, in small cell lung carcinoma EGCG increased caspase-3 activity [48]. Moreover, EGCG increased Bax/Bcl-2, p53, p21, caspases-3, and -9, PTEN and down-regulated PI3K, AKT, and Bcl-2 in T47D and HFF cells [49]. EGCG was also found to stimulate p21 and p27 expression in MDA-MB-468, MDA-MB-231 and HS578t cells, moreover, it inhibits Ki-67 in (PRB)-E2F/DP pathway [50]. Antiproliferative properties of EGCG were conducted on human esophageal squamous carcinoma cell lines in vitro and tumor xenografts in vivo. EGCG was found to exert cell cycle arrest at G_1_ phase in Eca-109 and Te-1 cells while the apoptosis was accompanied by elevated levels of ROS and caspase-3 cleavage. Considering in vivo experiment, proliferation was inhibited via decrease in VEGF [51]. Moreover, in HT-29 cells EGCG induced apoptosis due to Akt, ERK1/2 or p38 MAPK inhibition [52]. Furthermore, pro-apoptotic effects of EGCG were demonstrated in 293T cells via modulation of Bax/Bcl-2, caspases, and cytochrome c release [53]. A dose-dependent cytotoxic effect of EGCG was observed in A549 cells due to the reduction of Bcl-xL [54]. Gupta, S. et al., reported that EGCG decreased cell proliferation in induced apoptosis via inhibition of the NF-κB/p65 nuclear translocation [55,56]. In MCF-7 breast cancer cells EGCG inhibited the phosphorylation of ErbB2 and ErbB3 and suppressed the MAPK pathway [55,57], while in mammary tumor NF639 and SMF cells EGCG reportedly decreased cell proliferation, phosphorylation of ErbB2/neu and inhibited the NF-κB and MAPK pathways [55,58]. In SW837 colon cancer cells EGCG reportedly reduced the cellular levels of EGFR, ErbB2 and ErbB3 [55,59].

#### 3.2.2. Flavonoids (B Ring Attached to C2)-Unsaturated C-Ring

##### 3.2.2.1. Flavonol

**Figure 8:**
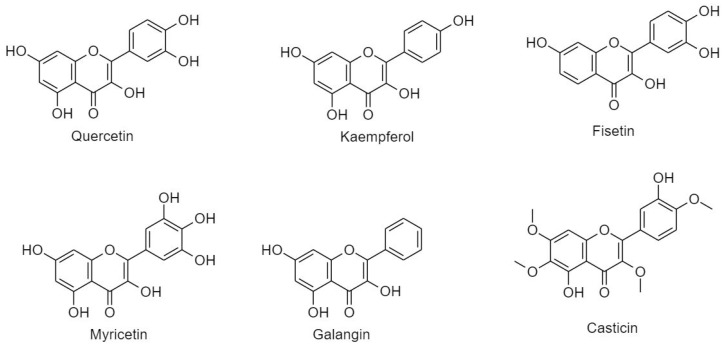
Classification and structure of flavonols.

Flavonol is characterized by the unsaturated C ring at C2-C3, which is found oxidized at C4 and hydroxylated at C3 [20]. Flavonol is widespread in kale, lettuce, onions, grapes, and berries. Flavonols are classified into six groups (Figure 8).

(A) Quercetin

3,5,7,3′,4′-Pentahydroxyflavone or quercetin is the most abundant flavonoid, widely distributed in barks, flowers, and seeds of tomatoes, apples, berries, grapes, onions, tea leaves, Brassica vegetables, capers, shallots, and nuts [26,27]. Quercetin is a phytoestrogen structurally resembling endogenous estrogen 17_β_-estradiol [60]. Several studies support the anticancer potential of quercetin. In MCF-7 cells, quercetin showed the tendency to induce both apoptosis via increase of Bax, capsase-3 and decrease in Bcl-2 and necroptosis through overexpression of RIPK1 and RIPK3 [61,62]. Another study demonstrated the ability of quercetin to induce intrinsic mitochondrial apoptotic pathway [63]. On the contrary, quercetin enhanced extrinsic apoptosis in the BT-474 cell lines. Interestingly, it had no effect on Bcl-2 or Bax and therefore did not initiate any intrinsic apoptotic pathway [64].

Mitochondrial-induced pro-apoptotic effects of quercetin were demonstrated in HL-60 cell lines via modulation of Cox-2, caspase-3, Bax, Bad, Bcl-2, cytochrome c, and PARP [65]. In BC1, BC3 and BCBL lymphoma cells, quercetin induced apoptosis via downregulation of PI3K/AKT/mTOR and STAT3 pathways [21,66]. Quercetin in combination with EGCG reduced cell viability via inhibition of MDR1 in PC-3 and LNCaP cells. Moreover, effective reduction of LNCaP over PC-3 suggests that quercetin also mediates p53 apoptotic pathway [23,67]. In a similar manner when co-administered with metformin, quercetin inhibited the proliferative PI3K/AKT pathway [68]. While quercetin decreased ErbB2 tyrosine kinase activity and the phosphorylation of PI3K and AKT om SKBR3 breast cancer cells [55,69], it reduced the phosphorylation of ERK1/2 and AKT phosphorylation and inhibited the NF-κB pathway in HepG2 liver cancer cells [55,70].

(B) Kaempferol

Kaempferol or 3,4,5,7-tetrahydroxyflavone is a nontoxic dietary flavonoid distributed in tea, kale, beans, onions, tomatoes, strawberries, broccoli, cabbage, apples, and grapes [71,72]. Kaempferol possess antioxidant, anti-inflammatory, antimicrobial, neuroprotective, and anticancer abilities. Kaempferol structurally resembles estrogen, thus it could have dual estrogenic/antiestrogenic effects depending on its concentration, and could be a candidate in treatment of hormonal based cancers such as ovarian, breast, and cervical; hepatocellular carcinoma; acute promyelocytic leukemia; and glioma [71,73,74]. In breast cancer MDA-MB-453 cells kaempferol induced DNA fragmentation and upregulation of p53 expression and phosphorylation leading to disrupting cell proliferative signaling, hence apoptosis [75]. Kaempferol was associated with the induction of intrinsic apoptosis in the A2780/CP70, A2780wt and OVCAR-3 cell lines via mitochondrial pathway in which increase of caspase-3, caspase-7, p53, Bax, Bad, and decrease in Bcl-xL was included. Kaempferol also decreased the proliferative signaling of Akt pathway and prevented angiogenesis via downregulation of VEGF [76]. Similar effects were observed in the case of HL-60 and NB-4 cells [77]. In another study, kaempferol synergistically increased the effect of cisplatin through overexpression of c-Myc in OVCAR-3 [78]. In HepG2 cells, kaempferol was found to induce apoptosis via ER stress and CHOP pathway [79]. Moreover, kaempferol enhanced apoptosis via mitochondrial membrane depolarization and down regulation of the expression of anti-apoptotic proteins x-IAP and survivin in A172 cells [80]. Additionally, in HeLa cell lines, kaempferol was associated with modulation of mitochondrial function and apoptosis via elevating the Bax/Bcl-2 ratio [81].

(C) Fisetin

3,7,3′,4′-Tetrahydroxyflavone, also known as fisetin, can be found in strawberries, apples, persimmons, grapes, onion, and cucumber. Fisetin is reported to possess neurotrophic, anticarcinogenic, and anti-inflammatory properties [82]. Fisetin showed the tendency to induce apoptosis in the LNCaP cell lines via the induction of PARP cleaving, release of cytochrome c, cleavage of caspases-3, -8, -9, increase of Bad, Bax and Smac/Diablo and downregulation of Bcl-2, Bcl-xL and x-IAP [83]. In DU145 and PC3 cells fisetin decreased the activity of NF-κB. In addition, the co-treatment of cancer cells with TRAIL and fisetin caused significant activation of caspases-3 and-8 and disruption of the mitochondrial membrane [84]. Similarly, fisetin was associated with reduction of cell viability and induction of apoptosis in SNU-1 cell lines [85]. Fisetin exhibited similar effects in HeLa cells both in vitro and in vivo via modulation of ΔΨm, caspases-3, -8, and Bax/Bcl-2 ratio leading to cleavage of PARP. Moreover, pro-apoptotic abilities were associated with fisetin in combination with sorafenib in xenografts [86,87]. Interestingly, fisetin induced ER stress mediated apoptosis in NSCLS [88]. Significantly, combinatorial treatment of fisetin and melatonin in MeWo and Sk-mel-28 cells resulted in cleavage of PARP protein, mitochondrial release of cytochrome-c, and inhibition of COX-2 and NF-κB proteins [89]. Furthermore, in U266 cells fisetin increased sub-G_1_ population, caspase-3 activation, over expression of Bax, Bim, and Bad; downregulation of Bcl-2 and Mcl-1; and continuous generation of ROS [21,90].

(D) Myricetin

3,5,7,3′,4′,5′-Hexahydroxyflavoneis or myricetin is a common dietary flavonoid found black tea, green tea, or wine. Myricetin is associated with antibacterial, antiviral, antioxidant, and anti-carcinogenic activities [91]. Protective role of myricetin against cancer was demonstrated in HCT-15 cells by DNA condensation, suppression in Bcl-xL and Bcl-2 expression, and increase in the release of mitochondrial AIF. However, no effect on the cleavage of caspases-3 and -9 was observed [92]. In UVB induced HaCaT cells, myricetin exerted pro-apoptotic effects via inhibition of Akt pathway. Decrease in the Bad phosphorylation, ΔΨm and release of cytochrome c, Smac, and AIF were also observed [93]. Furthermore, in SKOV3 cells myricetin induced nuclear chromatin condensation and fragmentation and DNA double strand breaks in dose-dependent manner [94]. In cisplatin resistant OVCAR-3 and A2780/CP70 cell lines myricetin overcame cancer chemoresistance and exhibited higher cytotoxicity than cisplatin. The underlying mechanism involved both intrinsic and extrinsic apoptotic pathways confirmed by regulated expression of Bcl-2 and DR5 respectively [95]. Finally, the mechanism of myricetin induced apoptosis in TNBC was comprehended by the induction of ROS that resulted in DNA double strand breaks, and activation of mitochondrial apoptotic pathway [96].

(E) Galangin

3,5,7-Trihydroxyflavone also known as galangin is highly concentrated in honey and propolis, and in the Chinese herb, lesser galangal. Findings of several studies suggested anticancer properties of galangin. The effect of galangin was tested on male and female human colon cancer cell lines HCT-15 and HT-29 respectively. Results illustrated caspase and mitochondrial dependent apoptotic pathways including DNA condensation, activation of caspases-3 and -9, release of AIF, and loss of mitochondria membrane potential [97]. In SNU-484 cells galangin induced apoptosis and inhibited cellular proliferation in both dose- and time-dependent manner. Changes in cell morphology, PARP cleavage, DNA fragmentation, activation of caspases-3 and -9, and MAP kinase pathway were all observed in treated cells [98]. In HepG2, Hep3B, and PLC/PRF/5by cell lines, galangin showed pro-apoptotic tendencies through translocation of Bax to the mitochondria, decrease in Bcl-2 expression, activation of caspase-8 and Bid, release of cytochrome c, and PARP cleavage [99,100]. Moreover, pro-apoptotic effects of galangin in B16F10 cells were associated with disruption of ΔΨm cleavage of procaspases-3 and -9 and PARP and p38 MAPK pathway [101]. Furthermore, galangin also reversed TRIAL resistance in Caki, ACHN, and A498 cells by downregulation of Bcl-2, c-FLIP, Mcl-1, and survivin, and inhibition of NF-κB pathway. Galangin in combination with berberine synergistically led to cell cycle arrest in Eca9706, TE-1 and EC109 cells at G_2_/M phase [102].

(F) Casticin (vitexicarpin)

3′,5-Dihydroxy-3,4′,6,7-tetramethoxyflavone, or casticin, is well known for its strong pharmacological profile and use as an anti-inflammatory, neuroprotective, analgesic, immunomodulatory, and most importantly estrogenic and anti-carcinogenic agent [103,104]. In vitro investigation on human NSLC cell lines H460, A549 and H157 showed 50% reduction of cell viability associated with membrane depolarization, mitochondrial release of cytochrome c, activation of caspases-3 and -9, increase of DNA fragmentation, downregulation of x-IAP and Bcl-xL proteins, and upregulation of Bax and Bid [105]. In BGC-823, SGC-7901, and MGC-803 cell lines and in human colon cancer HT-29, HCT-116, SW480, casticin induced apoptosis intrinsically by down regulation of c-FLIP, Bcl-2, x-IAP, and survivin. Extrinsic pathway was potentiated by TRAIL-induced cytotoxicity as it intensely upregulated DR5 receptor expression with no effects on DR4 or decoy receptors [106]. Treatment of PANC-1 cells and PC-3 cell lines with casticin led to the induction of apoptosis and cell cycle arrest via altering Bax/Bcl-2 ratio and activation of caspase -3 [107,108]. Similar effects were observed in U251, U87, and U373 cells [109,110]. In xenografts of NOZ and SGC996 cells, casticin inhibited proliferation, induced apoptosis, and caused cell cycle arrest at G_0_/G_1_ via mitochondrial apoptotic pathway in which modulation of Bax, Bcl-2, cyclin D, caspase-3, -9, and PARP were observed [111].

##### 3.2.2.2. Flavones

**Figure 9:**
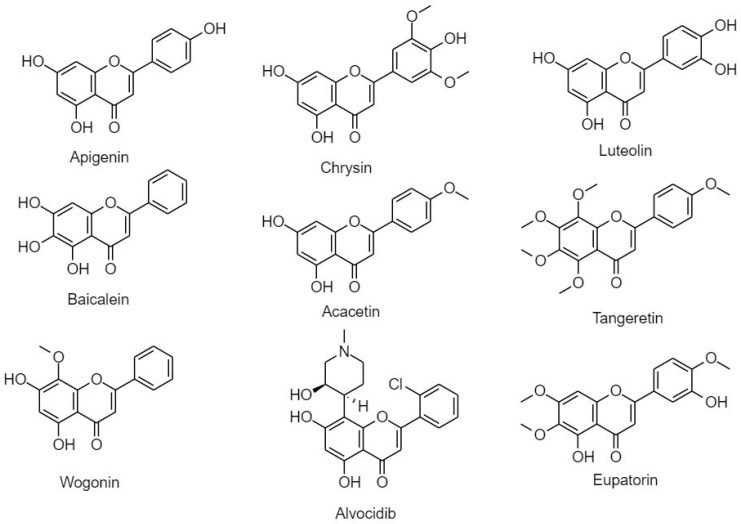
Classification and structure of flavones.

Flavones are distributed mainly in leaves, flowers, and fruits. Celery, parsley, mint, chamomile, red peppers, and ginkgo biloba are considered to be rich sources of flavones [20]. Similar to flavonols, their structure is comprised of an unsaturated C ring at C2-C3 position and a ketonic group at C4, but unlike flavonols they lack hydroxylation at C3 [20]. Flavones include apigenin, chrysin, luteolin, baicalein, tangertin, acacetin, flavopiridol/alvocidib, wogonin, and eupatorin (Figure 9).

(A) Apigenin

Apigenin or 5,7,4′-trihydroxyflavone is a phytoestrogen aglycone abundantly found in vine spinach, oranges, garlic, parsley, celery, carrot, propolis, artichokes, oregano, and chamomile. Apigenin anticancer efficacy in PC-3, 22Rv1, and DU145 cells resulted from the inhibition of HDAC enzymatic activity on transcriptional and translational levels leading to hyperacetylation of H3 on the *p21/waf1* promoter. Correspondingly, Bax overexpression, knockdown of x-IAP, c-IAP1, c-IAP2 and survivin, Bcl-2 and Bcl-xL proteins, as well as the release of cytochrome c were also observed [112,113]. Moreover, in SCC25 and A431 cells, apigenin alone or in combination with 5-fluorouracil (5-Fu) or cisplatin led to cell cycle arrest at G_2_/M phase via augmenting levels of ROS, reducing glutathione, and inducing TNF-R and TRAIL-R [114]. Apigenin also suppressed ACHN, 786-0, and Caki-1 RCC cell proliferation in vivo through cell arrest at G_2_/M phase, DNA damage, and p53 upregulation [115]. In T24 cell line, apigenin inactivated PI3K/Akt pathway, cyclins phosphorylation of p53, p21 and p27, activated the caspase cascade, released cytochrome c, downregulated Bcl-xL, Bcl-2m Mcl-1 and upregulated Bax, Bad and Bak [116,117]. In SW480 xenograft model, apigenin induced alteration in expression of cyclin D1, BAG-1, Bcl-2, and FADD which led to apoptosis [118]. Moreover, in BCPAP cells, apigenin inhibited viability in a dose-dependent manner due to enhanced ROS and subsequent induction of DNA damage [119]. In HCT-116 cells, apigenin induced intrinsic, extrinsic, and ER stress-initiated apoptosis together with increase of ROS and decrease in mitochondrial membrane potential and Ca^2+^ generation. Apigenin upregulated protein expression of CHOP, DR5, BID, Bax, cytochrome c release, and caspase cascade -3, -8 and -9 [120]. Apigenin reportedly reduced ligand induced phosphorylation of EGFR and ErbB2 thereby impairing their downstream signaling and thus induces apoptosis in head and neck squamous carcinoma cells [121]. Additionally, apigenin inhibited the proliferation and survival of malignant mesothelioma cells in vitro, increased the intracellular production of reactive oxygen species and induced DNA damage [122]. The apigenin induced cell death was related to the increase in the Bax/Bcl-2 ratio, p53 expression, the activation of caspases 9 and 8 and cleavage of PARP-1 [122]. In an in vivo C57BL/6 mouse model of malignant mesothelioma transplanted with #40a cells, intraperitoneal administration of apigenin reduced the risk of tumor growth and increased median survival rates in the apigenin treated mice [122]. Shukla, S. et al., reported that apigenin treatment decreased cell proliferation, increased proportion of cells in G_0_/G_1_ phase and decreased the levels of Rb and p38 kinase [55,123].

(B) Chrysin

5,7-dihydroxyflavone, or chrysin, is a flavonoid abundantly found in Thai propolis and honey. Chrysin is an apigenin analogue with high therapeutic potential favorable to intestinal membrane transport. However, its low bioavailability due to rapid metabolism and excretion renders its use less beneficial when compared to other flavonoid compounds [124,125]. Chrysin demonstrated high potency as an aromatase inhibitor in addition to its well-known role as an anti-inflammatory, antioxidant, and cancer chemo-preventive agent [126]. Chrysin was reported to be the most potent flavonoid functioning in the reduction of cell viability and induction of apoptosis in HeLa cell lines via increased DNA fragmentation and induction of p38 and NF-κB/p65. In Bcl-2 overexpressing U937 cell lines, chrysin showed pro-apoptotic effects through activation of caspase-3 and increased degradation of PLC-1, in addition to downregulation of x-IAP and inactivation of Akt [126]. Moreover, TRAIL-induced apoptosis associated with chrysin was observed in A549 and HeLa cell lines. TRAIL-induced cell death was selectively induced via inhibition of STAT3 and knockdown of Mcl-1 [97]. TRAIL-induced cell death after chrysin treatment was also observed in HCT-116 and CNE1 cells [127]. Recently a fully elucidated mechanism was exploited in DU145 and PC-3 cells including loss of MMP, increase in ROS, ER stress, and suppression of PI3K [128]. In SP6.5 and M17 melanoma cultured cells, chrysin activated mitochondrial dependent apoptotic pathway via loss of membrane potential, cytochrome c release, and activation of caspases-3 and -9 but not -8 [129]. Chrysin alone or in combination with cisplatin in HepG2 and QGY7701 cancer cells induced intrinsic and extrinsic apoptotic pathways due to increased levels of p53, Bax, caspases-3, -8, and -9 and DR5 along with decreased Bcl-2 levels [130,131].

(C) Luteolin

Luteolin or 5,7,3′,4′-tetrahydroxyflavone, is a well-known chemo-preventive, anti-inflammatory, and a cytotoxic chemotherapeutic agent [21,132,133]. In HepG2 cells, luteolin increased concentration of cytochrome c, translocated mitochondrial Bax and Bak and activated JNK pathway. In a tumor xenograft model, luteolin activated AMPK and NF-κB signaling and the release of ROS [134,135]. In NSLC A549 luteolin activated JNK, increased Bax, and promoted cleavage of caspases-3 and-9. Moreover, luteolin induced cytotoxicity, increased expression of p53 and p21, decreased MDM4 protein expression, and activated caspases-3 and -9 in vivo [136,137,138]. In NCI–H4 60 cells treated with luteolin apoptosis was modulated intrinsically, extrinsically, and via ER stress [139]. Similarly, ER stress and elevation of ROS in vitro and in vivo were observed in U251MG and U87MG cells [140]. The combination of luteolin with chemotherapeutic drugs 5-fluorouracil or gemcitabine synergistically induced anti-proliferative activity as reflected by decreased protein expression of nuclear GSK-3b and NF-κB, and increased cytochrome c [141]. In PANC-1, CoLo-357, and BxPC-3 luteolin increased Bax/Bcl-2 ratio associated with an increase in caspase-3, cleavage of PARP, and inhibition of NF-κB [136]. The Co-administration of luteolin and paclitaxel in MDA-MB-231 cells in mice xenografts resulted in reduction of tumor size via an increase in activated caspases-3 and -8 and increased expression of Fas. Luteolin also inhibited metastatic breast cancer cell lines by reducing expression of VEGF [142,143]. Luteolin was reported to induce intrinsic and extrinsic apoptotic pathway in HeLa cells via enhancement of caspase-3 and -8 activation cascade; reduction of ΔΨm associated with a release of cytochrome c; inhibition of Bcl-2 and Bcl-xL expression; and upregulation of death receptors Fas/FasL, DR5/TRAIL, and FADD [144].

(D) Baicalein

5,6,7-Dihydroxyflavone, or baicalein, is an aglycone found abundantly in the root of S. baicalensis [145]. Baicalein is known for its extensive pharmacological anti-inflammatory, anti-cardiovascular, and anti neuro-degenerative effects. Its pro-apoptotic effect on cancer cells is both mitochondria and receptor mediated, accomplished mainly by modulation of Bcl-2 family [146,147]. In SCC-4 cells, baicalein elevated Bax and lowered Bcl-2, modulated the release of cytochrome c, and activated the caspase cascade. Moreover, baicalein increased DR5 and TRAIL at protein and transcription levels and also elevated the level of ROS and induced ER stress [147]. Similar anti-proliferative effects of baicalein were observed in PCa cells [148]. Furthermore, in BxPC-3 and PANC-1cell lines baicalein suppressed proliferation and metastasis through reduction of Akt and ERK activities [149]. In CRC HT-29 baicalein induced cell cycle arrest in the G_1_ phase, DNA fragmentation, chromatin condensation; decreased Bcl-2 and increased Bax expression; reduced MMP-9; and controlled Akt. In mice xenografts, baicalein modulated Akt and p53 dependent pathway and reduced expression of MMP 2/9 [150,151]. Baicalein treatment in SGC-7901 cells in vitro and in vivo through disruption of ΔΨm, modulation of Bax/Bcl-2 in a dose-dependent manner [152]. Furthermore, in MDA-MB-231 cells pro-apoptotic effects of baicalein were associated with disruption of mitochondrial potential, release of cytochrome c, activation of caspases-3 and -9, increased Bax/Bcl-2, and elevated p53 expression and elevated signaling of ERK/p38 MAPK pathway. In addition, baicalein significantly inhibited invasion and metastasis by reducing the expression of MMP-2/9 and downregulating MAPK [153]. Baicalein downregulated Bcl-2 and upregulated caspase-3, -8, Bax, and Fas/FasL in HeLa cells [154]. Additionally, baicalein induced ER stress; upregulated Bax; downregulated Bcl-2, and induced cleavage of caspases-3, -9, and PARP in HCC cells in vitro and in vivo [155]. Similarly, baicalein inhibited tumor cell proliferation and induced G_2_/M arrest in HCC J5 cells [153].

(E) Tangertin

5,6,7,8 4′-Pentamethoxyflavone, also known as tangertin, can be found in citrus peel oil of the tangerine and other citrus fruits [156]. Tangertin was found to reduce DMBA-induced oxidative stress in breast cancer Sprague–Dawley rats by decreasing antioxidant enzymes SOD, CAT, GPx, GST, GSH, ascorbic acid and α-tocopherol [157]. In addition, tangertin was found to cause cell cycle arrest at G_1_/S phase via p53/p21 up-regulation, and inhibited metastasis by suppressing MMP-2 and -9 and vascular endothelial growth factor [158]. In AGS, in a dose-dependent manner, tangertin induced apoptosis via p53 dependent mitochondrial dysfunction and Fas/FasL extrinsic pathway. Tangertin also suppressed p53 inhibitor PFT-α via upregulation of p53, caspases-3, -8, and -9 activity, and Bax, t-Bid, p53, Fas, and FasL [159]. Moreover, in U-87MG and LN-18 tangertin induced G_2_/M arrest and apoptosis by modulating PTEN and cyclin-D and cdc-2 mRNA and protein expressions [160]. Additionally, tangertin in combination with TRIAL in H1299 and H1975 cells downregulated anti-apoptotic Bcl-2, Bcl-xL, survivin, x-IAP, c-IAP1, and c-IAP2 and elevated expression of pro-apoptotic Bax and caspases-3, -8, and -9. In addition to a significant increase in expression of DR 4 and 5, induction of ER stress was mediated via upregulation of CHOP and elevation of ROS [161].

(F) Acacetin

Acacetin is 5,7-dihydroxy-4′-methoxyflavone extracted from safflower flowers. Acacetin is associated with anti-peroxidative, anti-inflammatory, anti-plasmodial, and anticancer effects [162,163]. In T cell leukemia Jurkat cells, acacetin induced apoptosis through alterations in nuclear and cell morphology; induction of caspases-3, -8, and -9 activity; release of Apaf-1 and cytochrome c; and an increase in Bax/Bcl-2 ratio in a time-dependent manner [164]. The study conducted on the pro-apoptotic effects of acacetin in chronic lymphocytic leukemia (CLL) B-lymphocytes xenografts and healthy B-lymphocytes proved direct targeting of mitochondrial pathway via increased release of cytochrome c, caspase -3 activation, increased concentration of reactive oxygen species (ROS); and MMP collapse [165]. Acacetin showed antimetastatic potential in DU-145 cells via inhibition of phosphorylation of p38 MAPK involved in the knockdown of expression of MMP 2 and 9, and u-PA at protein and mRNA levels. In addition, acacetin was found to influence chromatin condensation and ability to significantly deplete nuclear NF-κB, which is associated with downregulation of Bcl-2 and x-IAP and proliferative protein COX-2 [163,166]. Moreover, in MCF-7 and HSC-3 cell lines acacetin influenced growth inhibition, DNA fragmentation, and PARP cleavage; enhanced caspases-7, -8 and -9 activity; and decreased Bcl-2. Acacetin also decreased mitochondrial membrane potential, modulated release of AIF and cytochrome c, and enhanced ROS generation, which subsequently resulted in apoptosis [162,167].

(G) Flavopiridol/Alvocidib

Flavopiridol is a cyclin-dependent kinase inhibitor mainly targeting CDK 9 in phase II clinical trials. Flavopiridol has shown efficacy in the treatment of chronic lymphocytic leukemia (CLL) SU-DHL-4 and TMD8 cells. Generally, flavopiridol initiates cell cycle arrest and apoptosis in a p53-independent mechanism that involves the down regulation of anti-apoptotic Mcl-1 and x-IAP and induction of ER stress [168,169,170,171]. In HCC the inhibitory effect of flavopiridol was associated with Mcl-1 inhibition that enhanced caspase-3 activation and PARP cleavage [172]. Interestingly, doxorubicin applied in combination with flavopiridol in HCC mouse models significantly reduced proliferative signaling [173]. Moreover, the anti-proliferative and pro-apoptotic effects of the flavopiridol in combination with oxaliplatin in melanoma cells and its subcutaneously injected allograft were associated with enhanced caspase -3, -7 and -9 activities in a dose dependent manner via the mitochondrial apoptotic pathway [174]. In metastatic osteosarcoma U2OS, SaOS-2, SJSA-1, and 143B, at nanomolar concentrations flavopiridol treatment led to significant reduction of cadherin 3 and 4, thus reducing metastatic potential in lungs [175].

(H) Wogonin

Wogonin, or 5,7-dihydroxy-8-methoxyflavone, extracted from *Scutellaria baicalensi* is associated with antioxidant, anti-inflammatory, anticonvulsant, neuro-protective, anti-bacterial, and anti-viral effects. Wogonin in LNcaP cells elevated levels of p21, p27, p53 and PUMA, which are involved in apoptotic Bax signaling [176]. Interestingly, wogonin in SK-N-BE2 and IMR-32 cell lines influenced mitochondrial dysfunction and ER due to the release of cytochrome c; altered expression of Bcl-2, Bax, and Bid; activation of caspases-3, -4, -8, -9, and -12; increased PARP cleavage; and ER stress-related proteins GRP-78 and GRP-94 [177]. In NSLC A549 and HeLa cell lines synergetic effects of wogonin and cisplatin were observed via potentiation of caspase-3 activity, and subsequent cleavage of PARP and H_2_O_2_ accumulation [178]. The viability of HT-29 cells was suppressed in a dose-dependent manner after wogonin treatment via induction of cell cycle arrest at the G_1_ phase; increased DNA fragmentation, chromatin condensation, and Bax/Bcl-2 ratio, inactivation of PI3K/Akt pathway; and upregulation and activation in a p53 [151]. In A2780 cells wogonin inhibited proliferation and metastasis via reduction of VEGF, Bcl-2 and Akt expression, and augmentation of Bax expression and p53 caspase-3 cleavage [179] Cytotoxic effects of wogonin were observed in HCC cell lines and xenografts via inactivation of AKT signaling, leading to the elevated levels of H_2_O_2_ and ER release of Ca^2+^ [180]. HPCC cells treatment of wogonin led to apoptosis due to enhanced ROS generation [181].

(I) Eupatorin

5,3′-Dihydroxy-6,7,4′-trimethoxyflavone, also known as eupatorin, is isolated from *Orthosiphonstamineus*, leaves of *Lantana montevidensis,* and in the aerial plants of *Tanacetum vulgare*. Eupatorin showed inhibitory effects on MDA-MB-468 cell lines at sub micromolar concentrations and in a dose dependent manner. Eupatorin led to cell cycle arrest at G_2_/M phase. The mechanism was elucidated by the selective activation of metabolic pathway of CYP1 to a cytotoxic active ingredient [182]. Similarly, induction of cell cycle arrest at G_2_/M phase was also reported in HeLa cells. In addition to activation of BAX in a p53 independent pathway, Eupatorin upregulated p53, p21 caspases-3 and -7, and PARP cleavage [183]. In HT-29 and SW948 cell lines, eupatorin decreased cell viability and induced apoptosis via a decrease in mitochondrial membrane potential, accompanied by an increase in Bax/Bcl-2 expression and an increase in ROS levels [184]. Treatment of eupatorin in combination with doxorubicin in colon cancer simultaneously increased the Bax/Bcl-2 ratio, caspase-3 expression, and PARP cleavage and induced apoptotic cell death [185]. In human leukemic cells it induced apoptosis via mitochondrial release of cytochrome c, activation of multiple caspases, and PARP cleaving [186]. Finally, the induction of apoptosis and cell cycle arrest in HepG2 was effectively induced via modulation of p53, MAPKs, and the mitochondrial pathways [187].

##### 3.2.2.3. Anthocyanidins

Anthocyanidins are characterized as a water-soluble, unsaturated, unoxidized class of flavonoids, occurring as plant pigments responsible for plant coloration depending on pH which contributes to their attractiveness.

**Figure 10:**
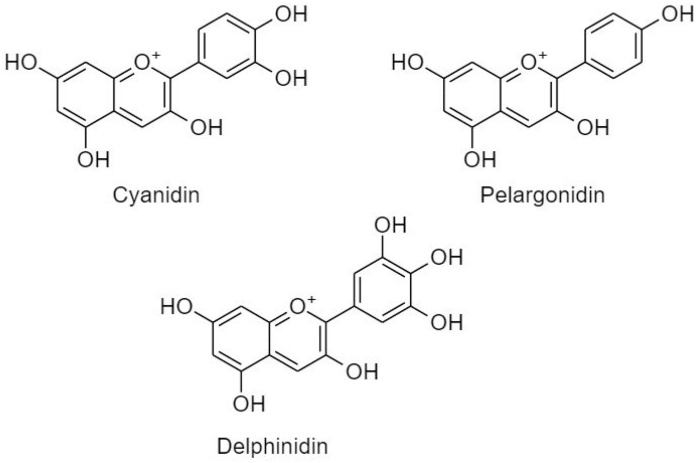
Classification and structures of anthocyanidins.

The antioxidant activity of anthocyanins is based on their phenolic structure and multiple hydroxylation in position 3 of ring C and in the 3′, 4′ and 5′ positions in ring B of the molecule (Figure 10). Anthocyanidins (Figure 10) are associated with reduction of DNA damaging oxidative adducts, diminishing lipid peroxidation, inhibiting mutagenesis, and modulating signaling cascades, thus reducing cellular proliferation. Aglycons are usually more potent antioxidants owing to fewer number of sugar groups [188,189] exhibiting wide pharmacological effects on cardiovascular disease and obesity, and antitumor, anti-inflammation, and anti-mutagenesis activities [190].

(A) Cyanidin

Cyanidin is usually found conjugated to a sugar, e.g., cyanidin-3-*O*-β-glucopyranoside (C3G). It has shown great effects in inducing cell cycle arrest, inhibiting proliferation (by impeding VEGF), metastasis (by the inhibition of MMP 2 and 9), and enhancing apoptosis caspase-independent AIF pathway (by activating cytochrome c and Bax protein expression) in various cancer cell lines [191]. Antiproliferative effect of C3G in DU145 and LnCap cell lines was mediated through activation of caspase-3 and induction of p21 protein expression, along with an increase in DNA fragmentation and levels of tumor suppressor P75NGFR [192]. In U87 cells, C3G showed pro-apoptotic effects via elevation of RNA expression of Bax and p53, whereas the expression of Bcl-2 was decreased [193].

Similarly, in MCF-7 cells, C3G mediated apoptosis via influencing morphological changes, surface blebbing, and nuclear condensation [194]. Cyanidin induced apoptosis in RCC by enhancing caspase-3, inhibited metastasis via inhibition of E-cadherin, and inhibited tumor growth of nude mice xenografts [195]. Cell cycle arrest at G_2_/M phase and DNA fragmentation were observed in U937 after cyanidin treatment [196]. Finally, cyanidin-3,5-diglucoside, cyanidin-3-glucoside, cyanidin-3-rutinoside, and peonidin-3-glucoside blocked SMMC-7721 cells in vitro and H22 cells in vivo via inducing cell cycle arrest at G_2_/M, DNA damage, increase in the activities of antioxidase, and a decrease in the level of lipid peroxidation [197].

(B) Pelargonidin

Pelargonidin is an anthocyanidin that is abundant in berries [198]. The acylated pelargonidin derivatives displayed a cytotoxic effect in Bel-7402 cells in a concentration-dependent manner associated with its antioxidant and pro-oxidant properties, along with influence on DNA damage [199]. Pelargonidin-glucoside in HT-29 induced apoptosis via activation of caspase-3 [188]. Correspondingly, pelargonidin exposure to HT-29 cells caused the release of cytochrome c from mitochondria; upregulated the activities of caspase-3 and -9, Bax, and Bid; downregulated the expression of Bcl-2 and Bcl-xL in addition to cleavage of PARP; and induced the expression of p53 and p21waf1 I [198]. Moreover, in U2OS cell line, pelargonidin triggered ROS-induced reduction in MMP and led to G_2_/M cell cycle arrest. In addition, pelargonidin also reduced the expression of proliferative cascades p-PI3K and p-AKT in a concentration-dependent manner [200].

(C) Delphinidin

Delphinidin is a major anthocyanidin found profusely in various colored fruits and vegetables, such as red cabbage, berries, sweet potatoes, and grapes. Delphinidin possesses anti-angiogenic, anti-inflammatory, anti-mutagenic, antioxidant, and anti-tumorigenic abilities [201,202]. In SKOV3 cells, delphinidin reduced proliferation in a dose dependent manner through inactivation of PI3K/AKT and ERK1/2 mitogen-activated protein kinase signaling cascades [202]. Similarly, in NSCLC delphinidin inhibited the activation of PI3K, phosphorylation of AKT and MAPKs; cleavage of PARP; activation of caspases-3 and -9; downregulation of Bcl-2, Bcl-xL, and Mcl-1; and upregulation of Bax and Bak. In mice xenografts significant inhibition of tumor growth along with an obvious decrease in cell proliferation markers Ki67, PCNA, and angiogenesis CD31, VEGF were also observed after the treatment [203]. Furthermore, in B CLL, delphinidin-3-*O*-glucoside and delphinidin-3-*O*-rutinoside demonstrated pro-apoptotic effects through a redox-sensitive caspase -3 activation mechanism as well as dysregulation of the Bad/Bcl-2 pathway, by rapidly dephosphorylating Akt and Bad and downregulating Bcl-2 [204]. Additionally, delphinidin induces apoptosis and cell cycle arrest in highly metastatic human prostate cancer PC3 cells and androgen refractory human prostate cancer 22Rnu1 cells through the inhibition of several components of NF-κB pathway [55,205,206]. Intra-peritoneal administration of delphinidin (2 mg), thrice a week, significantly reduced tumor growth in athymic nude mice implanted with PC3 cells (an in vivo model of prostate cancer). Investigations revealed that treatment with delphinidin significantly decreased the expression of NF-κB/p65, anti-apoptotic Bcl2, Ki67 and PCNA in these tumors [55,206].

#### 3.2.3. Isoflavonoids (B Ring Attached to C3)

**Figure 11:**
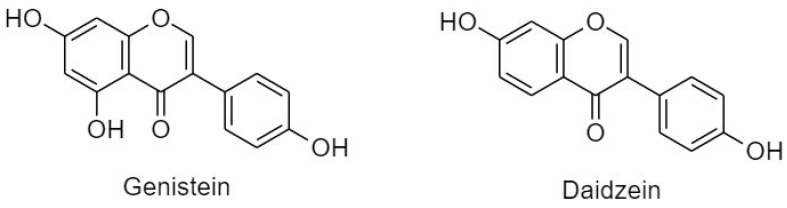
Classification and structure of isoflavonoids.

Distribution of isoflavonoids in plants is limited to soybeans and legumes, but they were also found in some microbes [20]. Isoflavonoids are further subdivided into genistein and daidzein (Figure 11). High consumption of isoflavonoids was correlated with decreased risk of estrogen related cancers [23] due to its estrogenic activity and ability to compete with 17_β_-estradiol in vitro and in animal models [20,73]. It was also confirmed that isoflavones exert DNA photoprotective effects and upregulates the expression of glutathione peroxidase enzyme [27].

(A) Genistein

Genistein or 5,7,4′-trihydroxyisoflavone, is a phytosterol found profusely in soybeans [207,208]. Genistein affected proliferation, differentiation, and apoptosis in MCF-7 and 3T3-L1 cells via regulating estrogen receptor-α (ERα) expression and altering Bax/Bcl-2 ratio [21,209]. Cytotoxic action of genistein against MDA-MB cells involved prooxidant signaling due to the mobilization of endogenous copper Notch-1 pathway which resulted in inhibition of NF-κB activity, and downregulation of Bcl-2 and Bcl-xL [210,211]. Moreover, in A2780, C200 cells genistein downregulated Bcl-2, Bcl-xL, c-IAP1, survivin, and NF-κB [212]. In LNCaP cells genistein induced apoptosis via augmented TRAIL-induced disruption of mitochondrial membrane potential [213]. The combination of Daidzein and genistein showed a synergistic effect on inhibition of cell proliferation and inducement of apoptosis in LNCaP and C4-2B [214]. Similarly, combination of genistein and selenium in PC3 and LNCaP (hormone-dependent) significantly inhibited growth and metastasis in dose- and time dependent manner by decreasing the expression of in MMP-2, which has been associated with active invasion and metastasis [215]. In HT-29 cell lines, the effect of genistein was explained by activating and dephosphorylating tumor suppressor FOXO3, which is usually attenuated by EGF [216]. The potency of genistein against HT-29 colon cancer showed that apoptosis was induced by increased caspase-3 activity at the transcriptional, protein, and enzymatic levels and reduced matrix metalloproteinase-2 (MMP2) activity [217] while it acted by inducing the mitochondrial pathway of apoptosis in HCT-116 and LoVo cells by inhibiting phosphorylation of Akt [218].

(B) Daidzein

7,4′-Dihydroxyisoflavone, also known as daidzein, is a dietary phytoestrogen isolated from soybeans. It is structurally similar to genistein; however, it lacks the hydroxyl group at position 5 [208]. Daidzein showed antiproliferative effects in different cancer cell lines. In MCF-7 cells and xenografts in a concentration- and time-dependent manner, daidzein stimulated production of ROS; release of cytochrome c from the mitochondrial membrane; activation of caspases-7 and -9; and alteration in Bax/Bcl-2 ratio [219,220]. Daidzein was also involved in induction of apoptosis in HCCSK-HEP-1, associated with up-regulation of Bak and down-regulation of Bcl-2 and B-xL proteins; release of mitochondrial cytochrome c; APAF-1; and activation of caspases-3 and -9 [221].

## 4. Final Remarks, Future Perspectives and Conclusions

Plant extracts have been proven to be strong candidates in the treatment of various types of cancer via modulating apoptotic pathway. The major mechanism involves the activation of apoptotic proteins intrinsically and extrinsically, elevation of ROS, and induction of DNA damage. The IC_50_ values of the various flavonoid compounds for various cancers discussed in the current manuscript is provided in Table 1.

### 4.1. Bioavailability

Bioavailability is the quantity of a compound that is absorbed and metabolized in the human body after it is ingested, and is commonly measured in terms of maximum plasma concentration (Cmax) [55]. Epidemiological data revealed an association between a diet rich in flavonoids and the prevention of human diseases. The efficacy of flavonoid compounds as cures and protective agents against a variety of human disease was tested extensively in in vitro experimental models and there remains a disparity between biological properties of flavonoids observed in vitro and their bioactivity in vivo [244,245,246]. Similarly, while many of the naturally occurring flavonoids have the potential to be effective anti-cancer agents in vitro such beneficial effects cannot be achieved in humans primarily owing to the low bioavailability of many of these plant-derived secondary metabolites in the body [244,245,246]. Hence the bioavailability of anti-cancer flavonoids must be investigated in detail. To explain the bioactivity of a flavonoid compound (the compound must enter the circulation and reach the tissues, in its native or metabolized form, in a sufficient quantity to exert biological activity) it is critical to understand the process of its absorption, bioavailability and its metabolism [244]. In the body glycosylated flavonoids such as flavonols, isoflavones, flavones and anthocyanins are generally hydrolyzed into their respective aglycones by intestinal or colon microflora prior to absorption [55,244,247]. The lipophilic aglycones enter the intestinal epithelial cells via passive diffusion while the epithelial transporters support the uptake of glycosides into the intestinal epithelial cells [247]. The rate of absorption of glycosylated flavonoids are much lesser when compared to than aglycone flavonoids in the intestine [245,248]. After absorption, flavonoids are subject to metabolic modifications primarily in the small intestine, liver and kidney [244,247]. The post-absorptive metabolic changes such as methylation, sulfation, or glucuronidation of the flavonoids before entering circulation and target tissues could also vastly alter their biological properties [55]. Additionally, the ingested flavonoid compounds, that remain unabsorbed in the proximal intestine, reach the colon where they are exposed to the colonic microbes which degrade these flavonoid molecules during which the heterocyclic oxygen containing ring is split and the subsequent degradation products (usually hydroxylated phenyl carboxylic acids) can then be absorbed [244]. Gallic acid and isoflavones, flavanones, catechin and quercetin glucosides are known to have the highest bioavailability while anthocyanins and pro-anthocyanidins have the lowest [246].

In precision medicine major progress in understanding the bioavailability of several flavonoids has been made, but practical solutions to overcome the poor oral bioavailabilities of many of the flavonoid compounds are still lacking [249]. Increasing bioavailability can be achieved by overstepping the barriers of solubility, cellular permeability, metabolic alteration and excretion of these compounds from the human body and facilitating target tissue uptake [249]. Several research groups are attempting to enhance the bioavailability of protective flavonoid compounds by improving intestinal absorption, altering the site of absorption and improving metabolic stability [250]. Although some of the flavonoid compounds are now commercially available in the form of pills, one must exercise caution because the use of large amounts of concentrated flavonoids may pose public health concerns owing to the limited information available on the adverse side effects and drug interactions of these compounds in an in vivo experimental setting and in clinical trials [249]. Although debatable, studies reported that genistein, a soy isoflavone, stimulates the growth and proliferation of MCF-7 breast cancer cells by enhancing the insulin-like growth factor pathway even in the absence of hormones estrogen or progesterone [249,251,252].

There are several different biomolecules (carbohydrates and proteins) in the biological system that have the potential to affect the bioavailability flavonoids [247]. Factors such as age, sex and genotype, in varying degrees can modulate the expression of bioactivity of these biomolecules which in turn influence the absorption, metabolism, circulating concentrations, tissue exposure and elimination of the ingested flavonoid compounds [247]. Therefore, it remains an even bigger challenge to factor-in considerations on age, sex, ongoing disease condition/s, current medications, habitual diet (and physiochemical properties of the food) and nature of the gut microbiome to understand the bioavailability and efficacy of flavonoid compounds as anti-cancer drugs in clinical trials [247]. In order to achieve the effective therapeutic doses used in preclinical studies importance must be given to improved and targeted drug delivery techniques so as to achieve maximum efficiency with minimal adverse side effects. Advances in nanotechnology-based drug delivery systems opens up better opportunities for increasing solubility, improving bioavailability and enhancing the targeting capabilities of flavonoids [253]. Current research should focus on designing suitable molecular carriers for the flavonoid drugs to target tissues. Studies using nanoparticle (liposomes, poly-ethylene glycol liposomes, nickel-based, lecithin-based and nanoribbon) carriers for quercetin were reported to be successful in terms of drug delivery into solid tumors in in in vitro and in vivo models of cancers of the central nervous system, lungs, colon, liver and breasts [253,254,255,256,257,258,259].

### 4.2. Combination Therapy

Plant extracts have been proven to be strong candidates in the treatment of various types of cancer via modulating apoptotic pathway. The major mechanism involves the activation of apoptotic proteins intrinsically and extrinsically, elevation of ROS, and induction of DNA damage. Additionally, flavonoid-polyphenol/flavonoid and flavonoid-classical anticancer drug combinations reportedly have shown improved efficacy in the treatment of cancer.

A combination of pterostilbene and quercetin effectively reduced cell growth and proliferation, decreased Bcl-2 expression and increased cell death while decreasing the metastatic potential of B16M-F10 melanoma cells in vitro and decreased tumor growth, and improved survival rates in an in vivo mice model bearing B16M-F10 melanoma cells [260]. Genistein and resveratrol in combination reportedly decreased tumor growth and IGF-1 expression in SV40 rats bearing prostate cancer xenografts [261]. Genistein and thearubigin in combination showed synergistic effects and significantly reduced cell proliferation and caused G_2_/M phase cell cycle arrest in PC-3 prostate cancer cells [262]. A combination therapeutic approach using quercetin and EGCG decreased cell proliferation, induced cell death and caused G_2_/M phase cell cycle arrest in PC-3 and LNCaP prostate cancer cells [67]. In this combination of quercetin and EGCG, quercetin improved the bioavailability of the green tea polyphenol and decreased its post-absorptive methylation both in vitro and in vivo [263]. In SCID mice bearing LAPC-4 prostate cancer cell xenografts the combination treatment using quercetin and EGCG was associated with a significant inhibition of tumor cell proliferation, decrease in androgen receptor expression, suppression of the PI3K/Akt signaling pathway and stimulation of apoptosis via increase in the Bax/Bcl-2 ratio [264]. In another study luteolin and EGCG in combination reportedly at low doses (at which single agents induces minimal apoptosis) had synergistic effects and increased apoptosis in in both head and neck and lung cancer cell lines [265]. The same study reported that the combination of luteolin and EGCG decreased tumor growth, inhibited Ki67 expression, increased cell death and reduced tumor growth in in vivo athymic mice models bearing xenografts of head and neck squamous cancer and lung cancer cells [265]. The synergistic effects of the combination of luteolin and EGCG was attributed to increased phosphorylation of p53 and p53 dependent effects on cleavage of PARP and caspase-3 [265]. In MDA-MB-231 triple negative breast cancer cells, a combination of curcumin and EGCG decreased cell proliferation, growth, and viability and caused G_2_/M cell cycle arrest while decreasing tumor volume in athymic nude mice implanted with MDA-MB-231 cells which correlated to the decreased in the levels of VEGF receptor-1 (VEGFR1) [266]. Ellagic acid, a naturally occurring antioxidant occurring in walnuts, cranberries and strawberries potentiated the effects of quercetin on p21waf1/cip1, p53, and MAP-kinases in vitro [267]. Combinations of ellagic acid and quercetin and quercetin and resveratrol decreased cell proliferation, increased cell death via the increase in caspase-3 activity and caused cell cycle arrest in MOLT-4 leukemia cells [268,269].

Suganuma et al., reported that EGCG in combination with tamoxifen or sulindac decreased the release of TNFα and decreased cell proliferation in PC-9 lung cancer cells and that EGCG stimulated the effects of celecoxib and decreased cell proliferation, increased the levels of GADD153 and phosphorylation of p38 in PC-9 and A549 lung cancer cells [270,271]. In another study, a combination of EGCG and celecoxib reportedly decreased cell proliferation, growth and viability of LNCaP, PC-3 and CWR22Rv1 prostate cancer cells and decreased tumor growth, increased the Bax/BCl-2 ratio, PARP cleavage and the expression of caspases-3 and -9 while decreasing the NF-κB activity and serum levels of PSA and IGF-1 [272]. Combinations of EGCG with other anti-cancer drugs such as paclitaxel/docetaxel, doxorubicin, cisplatin, gemcitabine and tasocitinib effectively reduced cell proliferation, increased cell death, reduced tumor growth and improved survival rates in in vitro and in vivo models of cancers of the prostate, breasts, ovaries, liver and pancreas [273,274,275,276,277,278]. Quercetin in combination with doxorubicin markedly reduced cell proliferation, DNA and protein synthesis and cell invasiveness in MCF-7 and MDA-MB-231 breast cancer cells [279]. In HeP2 laryngeal cancer cells, a combination of quercetin and cisplatin decreased cancer cell proliferation and induced cell death/apoptosis which was mechanistically attributed to the decrease in the phosphorylation of Akt, in the levels of Bcl-xL and Ki67 and the activity of HSP70 while increasing the phosphorylation of JNK, c-fos expression, the Bax/Bcl-2 ratio, ROS production, activity of caspases-8 and -9 and release of cytochrome c [280]. Combinations of genistein with other anti-cancer drugs such as cisplatin and gemcitabine, effectively reduced cell proliferation, increased cell death and reduced tumor growth in different in vitro and in vivo models of pancreatic cancers [281,282,283]. Isoflavone treatment in PC-3 prostate cancer cells increased the susceptibility of these cells to radiotherapy both in vitro and in vivo, reduced tumor growth and metastasis to para-aortic lymph nodes while increasing the expression of Bax and PARP cleavage and decreasing the levels of Bcl-xL and survivin [284,285,286].

Ongoing and completed clinical trials have reported the safety and efficacy of polyphenols as anticancer agents [55,287,288,289]. Clinical trials are also currently testing the flavonoid-flavonoid/polyphenol and flavonoid-anticancer drug combinations due to the promising pre-clinical data available on the use of such combinations [55]. A phase I study showed the efficacy, safety and tolerability of muscadine grape skin extract (which contains ellagic acid, quercetin, and resveratrol) in men with biochemically recurrent prostate cancer [290]. In breast cancer patients receiving radiotherapy administration of encapsulated EGCG (400 mg, thrice a day, orally) reduced the levels of VEGF and HGF in the serum and suppressed the activation of MMP-2 and MMP-9 and inhibited factors associated with the progression and metastasis of breast cancer [291]. Additionally, the exposure of MDA-MB-231 cells to the serum of breast cancer patients who received a combination of EGCG and radiotherapy evidently suppressed MDA-MB-231 cell viability, caused G_0_/G_1_ cell cycle arrest and induced cell death by apoptosis [291].

Details of completed and ongoing clinical trials using quercetin, genistein, EGCG and various other flavonoids in the treatment of different cancers can be found at https://clinicaltrials.gov.

The use of drug combinations over single drug applications should prove beneficial in (1) decreasing the dosage/concentration of the drug being used, (2) improving bioavailability, (3) overcoming the resistance that can acquired against a drug, (4) sensitizing the chemotherapy/radiotherapy resistant cancers to treatment, (5) reducing cancer cell metastasis and invasion, (6) reducing chances of relapse and (7) improving overall efficacy of cancer treatment. Additional studies are warranted in order to examine the bioavailability, efficacy, safety, tolerance and drug delivery options for flavonoids to be used in the treatment of cancers.

## Figures and Tables

**Table 1:** IC_50_ values of various flavonoids in different types of cancer.

Compound	Cancer Type/Cell Line	Time Point (h)	IC_50_	Reference
Hesperetin	Myeloid	24	500 ± 100 µM	[222]
Naringenin	Lymphoid	48	164.9 ± 21.83 µM	[222]
EGCG	Breast T47D	72	14.17 µM	[49]
	Prostrate LNCaP	48	0.4 µM	[223]
	Prostrate PC3	48	38.95 µM	[224]
	Lung	24	70 µM	[225]
	Bladder		70–80 µM	[226]
	Acute T-lymphoblastic leukemia (CCRF-CEM)		16.04 ± 1.56 µM	[227]
Quercetin	Bladder	48	876.9 ± 13.1 µM	[222]
Kaempferol	Bone	24	148.4 µM	[222]
Fisetin	Erythroid	48	15 ± 2 µM	[222]
Myricetin	Colon	72	68.0 ± 20.4 µM	[222]
Galangin	Liver	24	100.4 ± 17.0 µM	[222]
Casticin	Lung A549	48	0.4 µMol/L	[228]
	Actute T-lymphoblastic leukemia (CCRF-CEM)	24 & 72	0.28 ± 0.02 µM	[227]
	Leukemia K562	48	5.95 µM	[229]
	Leukemia Kasumi-1	48	15.56 µM	[229]
	Leukemia HL-60	48	4.82 µM	[229]
Apigenin	ER- breast	24	60.4 ± 15.8 µM	[222]
Chrysin	ER+ breast	48	82.5 µM	[222]
Luteolin	Lung	72	35.9 ± 9.3 µM	[222]
Baicalein	Stomach	72	64.3 µM	[222]
Tangertin	Melanoma	48	0.3 µM	[222]
Acacetin	Oral squamous HSC-3	24	25 µg/mL	[162]
	MCF7	24	26.4 ± 0.7 µM	[167]
	Melanoma		0.79 mM	[230]
Flavopiridol	Bladder RT4/RTI12	24–72	150–350 nM	[231]
	Bladder T24/SUP	24–72	1000 nM	[231]
	GCT	72	60–70 nM	[232]
	SKOV, MCF-7, HeLa	72	280–350 nM	[232]
	Esophageal	72	100–150 mM	[233]
	HNSCC tumor xenografts	10 weeks	5 mg/kg/day	[234]
	Hematopoietic SUDHL4	12	120 mMol/L	[235]
	Prostrate PC3	12	203 mMol/L	[235]
Wogonin	Ovarian	72	19.9 ± 1.2 µM	[222]
Eupatorin	HeLa		11.72 ± 2.86 µM	[236]
	K562		4.29 ± 1.35 µM	[236]
	MCF7		16.61 ± 5.56 µM	[236]
	RPMI8226		4.77 ± 0.51 µM	[236]
	HL-60		14.09 ± 0.55 µM	[236]
	MOLT		4.74 ± 0.43 µM	[236]
	MDA-MB-468	96	0.5 µM	[182]
	MCF-7	96	50 µM	[182]
Cyanidin	HL-60	48	31.6 µM	[237]
Pelargonidin	HL-60	48	85.2 µM	[237]
	Osteosarcoma U2OS		15 µM	[200]
	HepG2	24	33 µM	[238]
Delphinidin	HepG2	24	77 µM	[238]
	HL-60	48	10.9 µM	[237]
Genistein	Hepatocellular Hepa1-6	24	20 µM	[239]
	Pancreatic Mia-PaCa2	24	20 µM	[240]
	Pancreatic PANC-1	24	25 µM	[240]
	Pancreatic H6C7	24	120 µM	[240]
	Colorectal HCT 116	24	690 µM	[217]
	Colorectal HCT 116	48	135 µM	[217]
	Colorectal HCT 116	72	61 µM	[217]
Daidzein	Colorectal HT-29	48	200 µM	[241]
	MIA PaCa-2	48	200 µM	[241]
	Ovarian SKOV3 [224]	24	20 µM	[242]
	BEL-7402	48	59.7 ± 8.1 µM	[243]

## Abbreviations

ΔΨm	Mitochondrial Membrane Potential
5-FU	5-Fluorouracil
AIF	Apoptosis Inducing Factor
Akt	Protein Kinase B
AMPK	Adenosine Monophosphate-Activated Protein Kinase
APAF-1	Apoptosis Protease Activating Factor 1
Bad	Bcl-2 Associated Death Promoter
Bak	Bcl-2 Antagonist and Killer
Bax	Bcl-2 Associated X Protein
Bcl-2	B Cell Lymphoma 2 Proteins
Bcl-xL	B Cell Lymphoma-Extra Large
BH3	Bcl-2 Homology 3
Bim	Bcl-2-Like Protein 11
BIR	Baculoviral IAP Repeat Domains
CAT	Catalase
c-Flip	Caspase FLICE-Like Inhibitory Protein
CHOP	C/EBP Homologous Protein
c-IAP	Cellular Inhibitor of Apoptosis Protein
Cox-2	Cyclooxygenase 2
DISC	Death-Inducing Signaling Complex
DR	Death Receptor
EGCG	Epigallocatechin Gallate
EndoG	Endonuclease G
ER	Endoplasmic Reticulum
ESC	Esophageal Squamous Carcinoma
FADD	Fas-Associated Protein with Death
FasL	Fas Ligand
GPx	Glutathione Peroxidase
GRP	Glucose Related Protein
GSH	Glutathione
HCC	Hepatocellular Carcinoma
HGF	Hepatocyte Growth Factor
Her2	Human Epidermal Growth Factor
HSP	Heat Shock Protein
MAPK	Mitogen-Activated Protein Kinase
Mcl-1	Myeloid Cell Leukemia 1
MDR	Multi Drug Resistance
MMP	Matrix Metalloproteinase
MOMP	Mitochondrial Outer Membrane Permeabilization
mTOR	Mammalian Target of Rapamycin
NAC	N Acetyl Cysteine
NF-κB	Nuclear Factor Kappa Beta Pathway
N-IAP	Neuronal Apoptosis Inhibitory Protein
NSLC	Non-Small Lung Carcinoma
Omi/HtrA2	Temperature Requirement Protein A2
PARP	Poly (ADP-Ribose) Polymerase
PI3K	Phosphatidylinositol 3-Kinase
PUMA	P53-Upregulated Modulator of Apoptosis
RCC	Renal Cell Carcinoma
ROS	Reactive Oxygen Species
Smac/DIABLO	Second Mitochondrial Derived Activator of Caspase/Direct Inhibitor of Apoptosis-Binding with A Low Isoelectric Point
SOD	Super Oxide Dismutase
t-Bid	Truncated-Bid
TNF	Tumor Necrosis Factor
TRADD	TNF-Related Apoptosis-Inducing Ligand
TRIAL	TNFRSF1A-Associated Via Death Domain
uPA	Urokinase Plasminogen
VDAC	Voltage Dependent Anion Channel
VEGF	Vascular Endothelial Growth Factor
x-IAP	X-Linked Inhibitor of Apoptosis
